# Metabolomics characterization of colostrum in three sow breeds and its influences on piglets’ survival and litter growth rates

**DOI:** 10.1186/s40104-018-0237-1

**Published:** 2018-03-07

**Authors:** Gianfranco Picone, Martina Zappaterra, Diana Luise, Alessia Trimigno, Francesco Capozzi, Vincenzo Motta, Roberta Davoli, Leonardo Nanni Costa, Paolo Bosi, Paolo Trevisi

**Affiliations:** Department of Agricultural and Food Sciences (DISTAL), Alma Mater Studiorum - University of Bologna, Viale Fanin 46, 40127 Bologna, Italy

**Keywords:** Colostrum, ^1^H–NMR spectroscopy, Metabolome, Pig breeds, Piglets survival

## Abstract

**Background:**

Colostrum is the first secretion produced by mammary glands during the hours immediately preceding and succeeding parturition. This secretion differs from milk and represents an essential vehicle of passive immunity, prebiotic compounds and growth factors involved in intestinal development. Most of the literature concerning colostrum composition refers mainly to human and cow; and little is known about pig colostrum metabolome and how it varies between pig breeds and different farrowing parity. Thus, the aim of the present research is to provide new information about pig colostrum composition and the associations between metabolites, the sows’ breed and the survival and growth rates of their litters.

**Results:**

Colostrum samples were gathered from 58 parturitions of sows belonging to three different breeds chosen for their importance in Italian heavy pig production: 31 Large White, 15 Landrace and 12 Duroc respectively. The defatted and ultrafiltered colostrum samples were analysed using ^1^H–NMR spectroscopy. Principal Components Analysis (PCA) was assessed on the obtained spectra. In addition, using a Stepwise Regression and a Linear Regression analyses the metabolites named after the signals assignment were tested for their associations with piglets’ performances. Twenty-five metabolites were identified, comprehending monosaccharides, disaccharides (such as lactose), organic acids (lactate, citrate, acetate and formate), nitrogenous organic acids (such as creatine) and other compounds, including nucleotides. PCA results evidence a clustering due to breed and season effects. Lactose was the main compound determining the assignment of the samples into different clusters according to the sow breed. Furthermore, some metabolites showed to be associated with piglets’ performance and survival traits: acetate and taurine were positively related to litter weight gain and piglets’ survival rate, respectively, while dimethylamine and cis-aconitate were linked to new-borns’ impaired ability to survive.

**Conclusions:**

The results obtained suggest that colostrum composition is affected by breed, which, together with environmental conditions, may cause changes in colostrum metabolites content with possible consequences on piglets’ performances. Among the identified metabolites, acetate, taurine, dimethylamine and cis-aconitate showed consistent associations with piglets’ survival rate and litter weight gain, implying that these compounds may affect new-borns’ ability to survive.

**Electronic supplementary material:**

The online version of this article (10.1186/s40104-018-0237-1) contains supplementary material, which is available to authorized users.

## Background

The pre-weaning litter environment has been proven to affect pigs’ development and performances during later life [1] and in particular colostrum intake, coupled with birth weight, was found to influence piglets’ growth and mortality [2–4]. Colostrum provides new-borns with energy and passive immunity [5, 6]: in particular, most of the literature concerns the effects of the different immunoglobulins on piglets’ health and survival capacities [7–9]. Studies on human and bovine colostrum suggested important roles in new-borns’ health also for other bioactive molecules, such as nucleotides, oligosaccharides, organic acids and peptides [10–12], but little is known about the presence of these metabolites in sows’ colostrum and their association with piglets’ performances. Furthermore, to date little or no information about pig breed influence on colostrum composition is available and most of the knowledge about metabolites composition of swine colostrum was produced on samples gathered after farrowing induction, that may alter colostrum composition [13]. In this study, 58 colostrum samples were collected during a natural parturition with the aims i) to analyse through a ^1^H–NMR-based metabolomics approach the colostrum compounds with a maximum 10 kDa molecular weight in three pig breeds, ii) to evaluate breed and season effects on the colostrum composition, iii) to test the associations between the identified metabolites, the sow reproductive performance, and the piglets’ survival and growth rates at day three after birth.

## Methods

### Animals and sampling

Colostrum samples were collected from 58 farrowings of pure breed sows: 12 from Duroc (D), 15 from Landrace (L) and 31 from Large White (LW) sows. The number of samples collected per breed reflected the different numbers of individuals reared in Italy for these three pig breeds. All sows were raised in the same commercial farm from May 2014 to August 2015, under the same indoor environmental conditions with an automated system to control temperature and relative humidity. Following the EU rules to guarantee pig welfare, from the fourth week post insemination, the sows were kept in groups of 10 and fed twice a day with 2.5 kg of the same flour mash diet (Table 1). Five days before the farrowing, the sows were moved into the farrowing room and housed in single cages, fed twice a day until farrowing with the same diet. Sows had free access to water along all the experimental period. For this trial, we have considered exclusively sows that were not treated with antibiotics and medical products during gestation and lactation periods.

**Table 1:** Ingredients and calculated composition of the sows’ diet expressed on a dry matter basis

	Units	Dry matter
Digestible energy	kcal/ration	3,320.76
	kcal/d	6,641.52
Ingredients		
Barley	%	42.00
Wheat bran	%	30.00
Wheat flour	%	11.00
Soya	%	7.00
Corn	%	4.30
Whole soybean	%	2.00
Calcium carbonate	%	1.63
Bicalcium phosphate	%	0.65
Fish oil	%	0.50
Sodium chloride	%	0.40
Mycotoxin binder	%	0.20
L-lysine monohydrochloride	%	0.15
Choline	%	0.11
Magnesium sulphate anhydrous	%	0.05
Threonine	%	0.05
Methionine	%	0.04
Composition		
Crude protein	%	16.48
Crude fat	%	3.70
Crude fiber	%	7.27
Starch	%	37.57
Starch + Sugar	%	41.03
Calcium	g	8.00
Available phosphorus	g	8.60
Digestible phosphorus	g	4.51
Available lysine	g	8.59
Digestible lysine	g	7.23
Available methionine	g	2.87
Digestible methionine	g	2.51
Methionine + Cysteine	g	5.99
Digestible methionine + Cysteine	g	5.06
Threonine	g	6.14
Tryptophan	g	2.03

Farrowing was not induced, and the colostrum sampling was carried out during natural parturition, after the birth of the first piglet and before the parturition of the last, across all teats. Furthermore, the sows that showed long parturitions or required farrowing induction were excluded from the sampling, in order to avoid possible confounds of colostrum variations. All samples were immediately frozen at − 20 °C and then stored at − 80 °C until the samples preparation for ^1^H–NMR analysis.

The parity, the date and the season of the farrowing and the reproductive performance data were recorded for each sow. The number of alive piglets and the litter body weight (LBW) were recorded at birth and at d 3, cleansed of the weight of the dead piglets. The litter weight gain (LWG) was then calculated for the period from birth to d 3. Furthermore, the number of weaners per litter was recorded as well as the occurrence of diarrhea during suckling (1 = presence of diarrhea events from piglets’ birth until weaning, 0 = absence of diarrhea event).

### Colostrum preparation for ^1^H–NMR analysis

Colostrum was thawed, carefully mixed by inversion, and 15 mL of each colostrum sample was diluted 1:1 with pure water. To each diluted sample, 0.02% of sodium azide was added, to inhibit bacterial growth during the sample preparation. Then the sample was defatted through a centrifugation at 4 °C for 30 min at 1,500 × *g*. The aqueous phase was transferred to a clean Falcon tube avoiding the outer layer of fat, and centrifuged again; this procedure was repeated three times. 5 mL of the obtained aqueous phase was then transferred in Amicon Ultra 10 kDa membrane centrifugal filters (Merck Millipore, Merck KGaA, Darmstadt, Germany) and filtered by centrifugation at room temperature for 90 min at 5,500 × *g*. This step was needed to eliminate immunoglobulins and other proteins with high molecular weight. The eluted sample was then weighted and lyophilized and stored in a dry environment at room temperature until analyses.

### ^1^H–NMR measurements

At the time of sample processing, for each milligram of the lyophilized sample, 250 μL distilled water was added. Eighty μL of the regenerated sample were centrifuged at 14,000×*g* for 5 min (Scilogex D3024 High Speed Micro-Centrifuge, Rocky Hill, CT, USA) and then added to 720 μL of distilled water and 100 μL of a D_2_O solution of 3-(trimethylsilyl)-propioniate-2,2,3,3-d_4_ (TMSP) (Cambridge Isotope Laboratories Inc., Tewksbury, MA, USA) with a final concentration of 6.25 mmol/L. ^1^H–NMR spectra were recorded at 298 K with an AVANCE spectrometer (Bruker BioSpin, Karlsruhe, Germany) operating at a frequency of 600.13 MHz, equipped with an autosampler with 60 holders. The HOD residual signal was suppressed by applying the NOESYGPPR1D sequence (a standard pulse sequence included in the Bruker library) incorporating the first increment of the NOESY pulse sequence and a spoil gradient. Each spectrum was acquired using 32 K data points over a 7,211.54 Hz spectral width (12 ppm) and adding 256 transients. A recycle delay of 5 s and a 90° pulse of 11.4 μs were set up. Acquisition time (2.27 s) and recycle delay was adjusted to be 5 times longer than the longitudinal relaxation time of the protons under investigation, which has been no longer than 1.4 s. The data were Fourier transformed and phase and baseline corrections were automatically performed using TopSpin software, version 3.0 (Bruker BioSpin, Karlsruhe, Germany). Signals were assigned through a combination of literature assignments and by the use of a multimedia library included in Chenomx NMR Suite 8.2 professional software (Chenomx, Edmonton, Alberta, Canada).

### Data analysis

Sows were grouped according to the parity order: from 1 to 3 (PO1; 27 sows) and parities equal to or higher than 4 (PO2; 31 sows). The parturition season was also taken into account and was assigned as follows: 1 = parturition between the 1^st^ of December and the 28^th^ of February; 2 = between the 1^st^ of March and the 31^st^ of May; 3 = between the 1^st^ of June and the 31^st^ of August; 4 = between the 1^st^ of September and the 30^th^ of November the average temperature per seasons registered was respectively 5.6 °C ± 0.9 °C for season 1, 16.5 °C ± 4.3 °C for season 2, 25.2 °C ± 4.3 °C for season 3 and 16.2 °C ± 4.2 °C for season 4. Among the studied animals, 6 sows gave birth during season 1, 19 during season 2, 21 during season 3 and 12 during season 4. The data collected about piglets’ performances were analysed using an analysis of variance (ANOVA) with the aim to identify possible differences linked to sows’ breed.

Statistical analyses on spectra data were performed using R computational language (ver. 3.1.2) [14] and MATLAB (ver R2014b, MathWorks Inc.). Each ^1^H–NMR spectrum was processed by means of scripts developed in-house as follows: spectrum baseline was adjusted by employing the signals identification algorithm named “baseline.peakDetection” from R (version 3.1.2) package “Baseline” (https://cran.r-project.org/web/packages/baseline/index.html).

Chemical shift referencing was performed by setting the TMSP signal to 0.00 ppm. The following spectral regions were removed prior to data analysis: the regions including only noise (the spectrum edges between 11.00 and 8.65 ppm and between 0.15 and − 1.00 ppm), the ^1^H–NMR signal which is strongly affected by the residual solvent signals (water, between 4.90 and 4.50 ppm) and the glycerol’s signals from 3.82 and 3.76 ppm, from 3.69 and 3.63 ppm and from 3.60 and 3.54 ppm**.** Spectra were then normalized by means of probabilistic quotient normalization method (PQN) [15] and binned. The first normalization operation is aimed at removing possible dilution effects. The second one avoids the effect of signals misalignments among different spectra due to variations in chemical shift of signals belonging to some titratable acids. The binning operation is performed by subdividing the spectra into 369 bins, each integrating 120 data points (0.0219 ppm each). In order to focus on the real information contained in the spectra, bins that had an average higher value than noise were selected. In this way, a total of 201 bins were kept.

The spectra obtained were then analysed through an unsupervised multivariate approach using PCA. The PCA was conducted on the 201 bins matrix to identify the outlier samples and test the existence variables contributing to samples clustering. The multivariate models were calculated and the results were visualized on both scores and loadings’ plot. In order to determine the spectral regions encompassing most of the discriminative information, bins with a loading value greater than 1% of the overall standard deviation of all loading values were selected. The identified metabolites were grouped in a new dataset named C-dataset. The C-dataset was used to conduct an ANOVA with the aim to confirm if the amounts of the identified compounds were influenced by the effects of breed and farrowing season identified with the PCA and parity order. The model utilized for this analysis was:

$$ y=\upbeta 0+{\upbeta \mathrm{p}}^{\times }b+{\upbeta \mathrm{p}}^{\times }s+{\upbeta \mathrm{p}}^{\times }o+{\upbeta \mathrm{p}}^{\times }n+{\upbeta \mathrm{p}}^{\times}\left({b}^{\times }s\right)+E $$

Where:

β0 was the intercept;

βp was the corresponding regression coefficient;

*y* was the amount of each identified metabolite;

*b* was the sow breed (LW; D; LA);

*s* was the farrowing season (1; 2; 3; 4);

*o* was the parity order (PO1; PO2);

*n* was the number of piglets born alive per litter;

*b*×*s* was the interaction between breed and season;

*E* was the error.

This first part was conducted to test if sows’ breed influences colostrum profile and if in addition to breed there are other “environmental” variables affecting colostrum quality (such as the farrowing season, the parity order or the litter size).

Then, a Stepwise Regression analysis was used to select, among the metabolites included in the C-dataset and sows’ reproductive performances, the variables that influenced the LWG, the number of dead piglets from birth until d 3 or the number of piglets dead from day 3 to weaning. This statistical analysis involves starting with no variables in the model and adding gradually each metabolite and sow reproductive parameter (the litter weight and the number of alive piglets at birth) to evaluate which one of the colostrum identified compounds and sows’ reproductive abilities most influenced the piglets’ survival and growth. The results obtained from the Stepwise Regression analysis were then confirmed through Generalized Linear Model (GLM). The GLM model for LWG included the sows’ breed, the average piglet’s weight at birth and the interaction between sows’ breed and acetate as fixed effects. The GLM model for the number of dead piglets from birth until day three included the sows’ breed, the number of alive piglets at birth, the interaction between sows’ breed and dimethylamine and the interaction between sows’ breed and taurine as fixed effects. For the number of weaned piglets the GLM model included as fixed factors the sows breed, the number of alive piglets at birth, the interaction between sows’ breed and cis-aconitate.

Finally, all the variables that did not show an effect on the dependent variables were removed from the model and only the significant effects were maintained.

The *prcomp* function of R environment was used to perform the PCA analysis on bins matrix [16]. The ANOVA analysis, the Stepwise Regression analysis, and the regression model were carried out on SAS software using PROC REG and PROC GLM respectively (SAS® 9.4, SAS Inst. Inc., Cary, NC). Results were considered significant at *P* ≤ 0.05 and tendencies at 0.05 ≤ *P* ≤ 0.10.

## Results

### Dataset description

Table 2 detailed the data about sows, litters and piglets included in the study. D sows had on average a lower number of piglets born alive per litter (8.92 ± 2.28) with respect to L (12.60 ± 1.72) and LW (11.90 ± 2.26) (*P* < 0.0001), while the new-borns of L and LW breeds presented a lower weight at birth (1.38 ± 0.15 kg and 1.43 ± 0.16 kg, respectively) compared to D piglets (on average 1.59 ± 0.23 kg) (*P* = 0.007).

**Table 2:** Phenotypic differences observed for the parameters measured in Duroc, Landrace and Large White litters

Variables	D^a^	L^b^	LW^c^	Total	*F* value	*P-*value
Number of sows	12	15	31	58		
Order of parturition	2.750	4.067	4.161	3.845	3.555	0.035
Number of piglets born alive per litter						
Mean	8.917	12.600	11.903	11.466	11.85	<.0001
SD	2.275	1.724	2.256	2.494		
Average LBW at birth^d^, kg						
Mean	14.033	17.207	16.890	16.381	5.503	0.006
SD	3.679	2.081	2.828	3.060		
Average piglet’s weight at birth, kg						
Mean	1.588	1.376	1.432	1.450	5.437	0.007
SD	0.226	0.153	0.155	0.184		
Number of alive piglets per litter at d 3						
Mean	8.250	12.133	10.871	10.655	12.55	<.0001
SD	1.865	1.767	2.291	2.453		
Number of dead piglets per litter at d 3						
Mean	1.600	1.750	1.583	1.619	0.104	0.902
SD	1.342	0.500	0.996	0.974		
Average LBW at d 3^d^, kg						
Mean	16.400	21.167	19.758	19.428	4.948	0.010
SD	3.993	3.059	4.264	4.211		
Average piglet’s weight at d 3, kg						
Mean	2.001	1.761	1.826	1.846	4.326	0.018
SD	0.275	0.240	0.213	0.244		
LWG^e^, kg						
Mean	3.085	4.453	4.103	3.983	0.102	0.902
SD	0.864	1.461	1.771	1.598		
Number of weaned piglets						
Mean	7.333	11.133	10.032	9.759	11.59	<.0001
SD	1.875	1.552	2.316	2.423		
Incidence of diarrhoea						
Mean	3	1	4	8	1.048	0.357

^a^Duroc

^b^Landrace

^c^Large White

^d^litter body weight

^e^the average litter body weight gain from birth to d 3

### Colostrum spectra

**Fig. 1:**
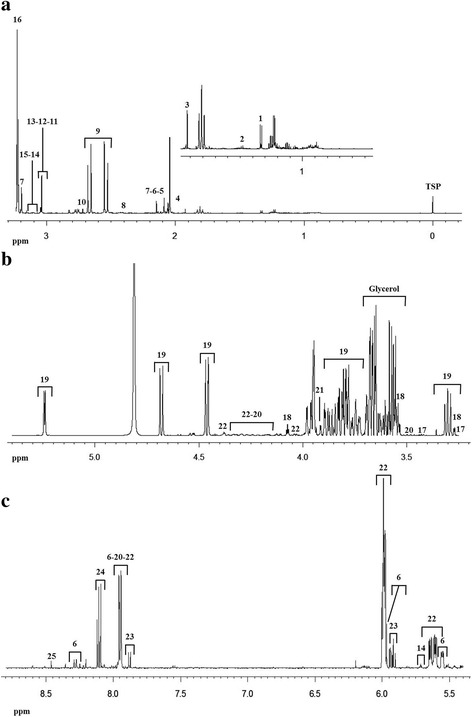
Typical ^1^H–NMR spectrum of aqueous extract of colostrum. The spectrum has been split into three parts for the sake of clarity. Some resonances have been assigned by using Chenomx software and listed in Table 3: **a** Aliphatic or upfield region; **b** Carbohydrate or midfield region, characterized by the presence of signals belonging to sugars and glycerol and **c** Aromatic region or downfield region

Figure 1 shows a ^1^H–NMR profile of defatted and ultrafiltered sow colostrum. The ^1^H spectrum is mainly dominated by the carbohydrate signals overlapping in the midfield region between 3.49 and 4.49 ppm (Fig. 1b). Those belong to lactose and nucleotides sugars such as UDP-glucose and UDP-galactose and nucleotide as UMP. Amino acids mainly fall in the upfield region, between 0.99 and 3.49 ppm, together with the signals of organic acids (Fig. 1a) and creatine (3.04–3.05 ppm). In this part of the spectrum fall also signals from threonine and lactic acid (both at 1.33 ppm), alanine (1.49 ppm), acetic acid (1.92 ppm), succinic acid (2.41 ppm) and citric acid (2.54 and 2.67 ppm). Finally, in the downfield region (Fig. 1c) signals of different phenolic compounds can be observed, but in this case, only formic acid was assigned (8.4 ppm), together with signals from the nucleotide sugars UDP-glucose and UDP-galactose (5.5–6 ppm, 7.9–8 ppm) and UMP (8.1 ppm, 5.98–5.99 ppm, 4.42 ppm) as listed in Table 3. The 25 compounds have been identified through a combination of literature assignments [17] and by the use of a multimedia library included in Chenomx NMR Suite 8.2 professional software (Chenomx, Edmonton, Alberta, Canada).

**Table 3:** Assignment table of the identified metabolites present in the ^1^H–NMR spectra of colostrum

Assigned number	^1^H chemical shift, ppm^a^	Compound
1	1.332 (d)	Lactate
2	1.486 (d)	Alanine
3	1.923 (s)	Acetate
4	2.028 (s)	N-Acetylglutamate
5	2.063 (s)	N-Acetylglucosamine
6	2.089 (s) - 5.552(dd) - 5.967 (d) - 7.944 (d) - 8.287 (d)	UDP-N-Acetylglucosamine
7	2.147 (s) -3.222 (s)	O-Acetylcholine
8	2.408 (s)	Succinate
9	2.539 (d) - 2.667 (d)	Citrate
10	2.720 (s)	Dimethylamine
11	3.039 (s)	Creatine
12	3.046 (s)	Creatine phosphate
13	3.050 (s)	Creatinine
14	3.119 (d) - 5.712 (m)	cis-Aconitate
15	3.204 (s)	Choline
16	3.231(s) - 4.330 (m)	sn-Glycerophosphocholine
17	3.272(t) - 3.532 (dd) - 4.073 (t)	Myo-Inositol
18	3.259 (t) - 3.428 (t)	Taurine
19	3.302 (t) -3.684:3.906 (m), 3.980 (d) 4.461 (d) - 4.679 (d) - 5.243 (d)	Lactose
20	3.480 (s) - 4.142:4.278 (m) -5.607 (dd) -5.967 (m) -7.940 (d)	UDP-glucose
21	3.935 (s)	Glycolate
22	4.142:4.278 (m) - 4.379 (m) - 5.664 (dd)- 5.990 (m) - 7.942 - 7.995(d)	UDP-galactose
23	5.917 (d) - 7.879 (d)	Uridine
24	8.406 (s)	Formate
25	4.423 (t) - 5.990 (m) - 8.102 (d)	UMP

The assignments were obtained at pH 7.420. Chemical shift values are referenced to TMSP proton signals at 0.00 ppm. Glycerol (3.568, 3.661 and 3.793 ppm has not been listed as it has not been included in the PCA)

^a^d, doublet; dd, doublet of doublets; m, multiplet; s, singlet; t, triplet

### Factors affecting colostrum composition

**Fig. 2:**
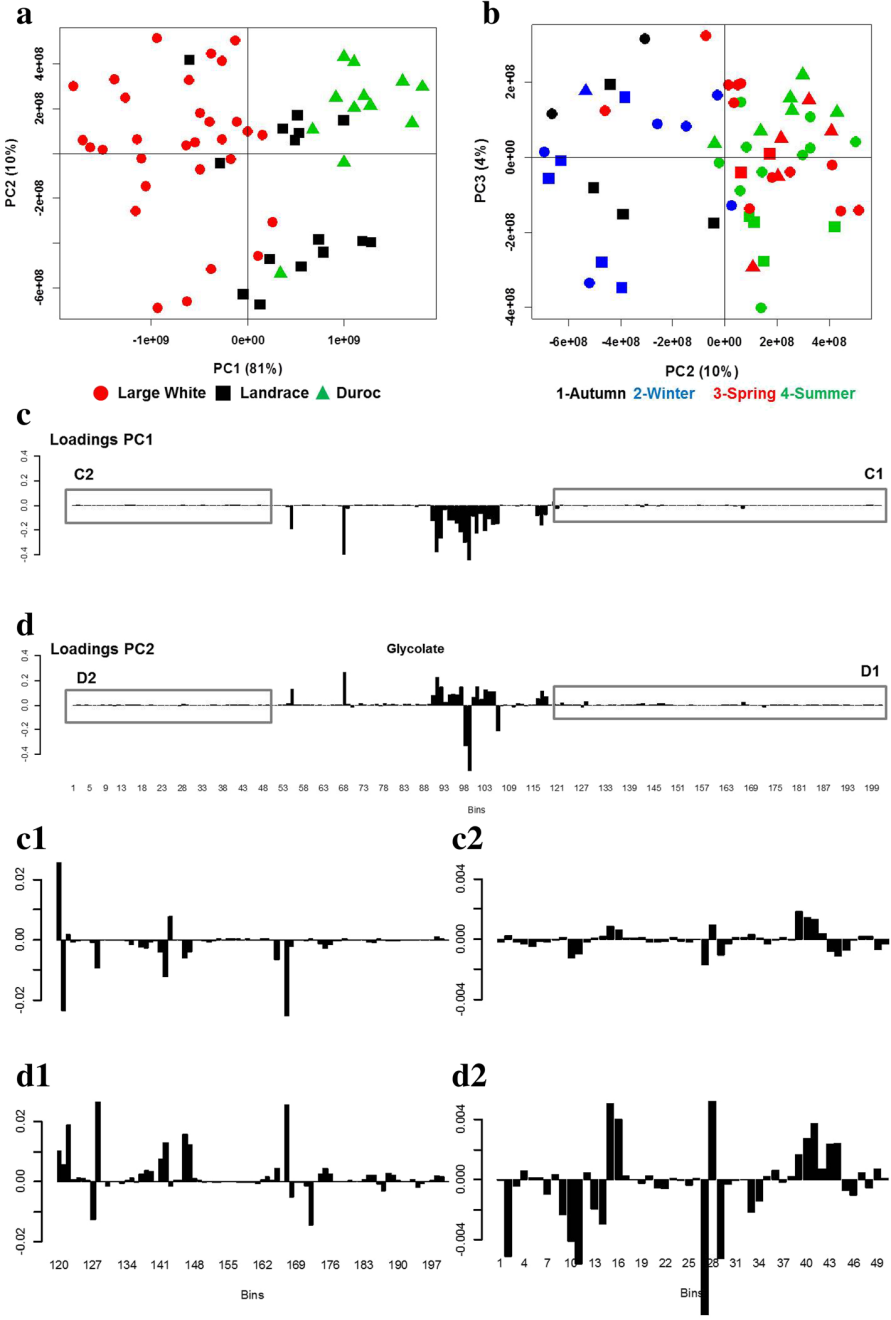
Score plots of PCA on ^1^H–NMR binned spectra of colostrum. **a** PC1 vs. PC2 and **b** PC2 vs. PC3. The first two PCs represent the 91% of the total variance. **c**-**d** Loadings bar-plot for spectral bins along PC1 and PC2 respectively. Downfield (C1 and D1) and upfield (C2 and D2) regions of C and D loadings bar-plot were expanded on the vertical scale to appreciate the presence of small bar plot

After alignment, normalization and binning, the dataset contained 58 colostrum spectra characterized by 201 bins and PCA was applied on it to investigate differences on the metabolome between groups. In the total colostrum spectra, no PCA clustering for sow’s parity order was identified (data not shown). Figure 2a and b show that samples clustered on PC1-PC2 due to the effect of the sow breeds (Fig. 2a) and on PC2-PC3 due to the farrowing seasons (Fig. 2b). The PC1 explained the 81% of the total variance and separated the colostrum spectra of D and LW, while PC2 (10% of the variance) discriminated the L colostrum composition into two clusters. The PC2 together with the PC3 explained the 14% of the total variance. This plot highlights the season effect, in particular along PC2 where differences in the colostrum spectra due to seasons 1 and 4 (winter-autumn) against season 2 and 3 (spring-summer) are visible. The weighting of each variable (bin) is represented by the loadings plot in Fig. 2c and d in which are displayed the loadings from PC1 and PC2 respectively as a bar plot, where each bar corresponds to a single spectral variable (bin). The main bins accounting for the spectral differentiation and their relative chemical shift were listed in the Additional file 1: Table S1 (SS1). As emerging from the SS1 table, most of the signals included in these discriminant bins were assigned to the corresponding metabolites. The C-dataset, which was used for the following statistical analyses, resulted as being composed of 25 metabolites, listed in Table 3.

The parity, breed and season effects on colostrum composition were then confirmed through the ANOVA analysis on the identified metabolites described in the C-dataset, and the results are reported in Table 4. Sows’ parity order and the interaction between sows’ breed and season did not show significant associations with the metabolites amount, while the number of piglets born alive showed few significant associations (*P* < 0.05 only for N-acetilglucosamine and UDP-glucose – data not shown). Table 4, reports the *P* values for breed and season, which showed the strongest effects on ultrafiltered colostrum metabolome. Indeed, the amounts of lactose, UDP-glucose, glycolate and UDP-galactose were strongly associated to breed (*P* < 0.001), citrate and N-acetilglucosamine showed breed-related differences (*P* < 0.01), as well as alanine, succinate, creatine, creatine phosphate, *cis*-aconitate, O-acetylcholine, sn-glycerophosphocholine, UDP-N-acetilglucosamine, taurine and myo-inositol (*P* < 0.05). In particular, the colostrum of L samples showed upper signals for UDP-glucose, UDP-galactose and sn-glycerophosphocholine compared to the other two breeds, while LW colostrum was characterized by a greater quantity of lactose, taurine, myo-inositol and glycolate than L and D colostrum. Season as well explained a significant part of the variations observed for acetate, dimethylamine, creatine phosphate, creatinine, *cis*-aconitate, glycolate and formate (*P* < 0.001), for creatine, taurine, UDP-galactose and UMP (*P* < 0.01) and for alanine (*P* < 0.05).

**Table 4:** Effects of sow breed and season on the identified colostrum metabolites

Metabolite	Breed^a^			SEM	*P-*value	Season^b^				SEM	*P-*value
	D	L	LW			1	2	3	4		
Lactate	5.38	6.7	8.88	1.85	0.506	4.93	10.18	8.71	4.13	1.81	0.495
Alanine	1.77	2.2	2.44	0.17	0.04	1.65	2.5	2.51	1.88	0.17	0.04
Acetate	9.57	11.17	9.9	0.91	0.378	13.59	7.55	5.95	13.77	0.89	<.0001
N-Acetylglutamate	6.35	9.9	14.03	3.77	0.453	7.94	9.69	8.53	14.21	3.69	0.776
N-Acetylglucosamine	10.9	15.4	11.7	1.6	0.003	10.6	14	13.8	12.3	1.6	0.149
UDP-N-Acetylglucosamine	22.4	34.4	33.9	2.2	0.014	26.2	33.4	33.8	27.4	2.1	0.324
O-Acetylcholine	77.1	196.9	156.6	13.9	0.002	101.7	148.4	171.7	152.3	13.6	0.115
Succinate	2.21	3.26	3.5	0.19	0.01	2.59	3.26	3.55	2.55	0.21	0.329
Citrate	209	301	257	13	0.002	246	286	265	228	12	0.101
Dimethylamine	2.86	4.44	4.51	0.49	0.091	2.3	5.19	5.39	2.85	0.48	0.0003
Creatine	39.8	59.9	58.7	3.4	0.014	40.9	59	64.7	46.6	3.3	0.002
Creatine Phosphate	3.4	7.99	8.01	0.88	0.015	1.9	8.5	10.78	4.69	0.86	<.0001
Creatinine	13.6	16.7	16.5	1.1	0.622	19.9	11.8	11.6	19.2	1.1	<.0001
*cis*-Aconitate	1.41	1.93	1.74	0.14	0.029	1.1	2.14	2.28	1.26	0.14	<.0001
Choline	7.82	10.62	9.91	1.19	0.665	8.86	11.64	9.43	7.87	1.16	0.961
sn-Glycerophosphocholine	446	543	414	38	0.038	430	457	507	477	37	0.507
Myo-inositol	63.53	76.95	82.86	3.76	0.012	62.58	73.17	81.13	80.91	3.68	0.085
Taurine	1.57	4.1	6.05	0.87	0.015	0.98	6.23	6.19	2.21	0.85	0.005
Lactose	458	579	811	30	<.0001	535	644	667	619	30	0.136
UDP-glucose	6.07	9.78	6.23	0.54	<.0001	6.08	7.3	8.09	7.97	0.54	0.198
Glycolate	28.1	39.8	45.8	2.4	0.0006	28.4	41.8	45.9	35.6	2.3	0.001
UDP-galactose	32.6	74	42.5	4	<.0001	39.3	54.2	61.4	44	3.9	0.005
Uridine	3.14	3.72	3.23	0.38	0.601	3.05	3.55	3.49	3.37	0.38	0.463
Formate	4.49	4.43	4.03	0.35	0.06	6.27	3.04	2.29	5.67	0.34	<.0001
UMP	13.3	24.2	21.6	1.7	0.096	21.82	16.04	16.84	24.08	1.91	0.01

Mean of the identified metabolites are expressed as absolute area

^a^The Breed is assigned as D for Duroc, L for Landrace and LW for Large White

^b^The seasons were assigned as follows: 1 = if the parturition was included in the period between the 1^st^ of December and the 28^th^ of February; 2 = between the 1^st^ of March and the 31^st^ of May; 3 = between the 1^st^ of June and the 31^st^ of August; 4 = between the 1^st^ of September and the 30^th^ of November

### Factors affecting litter performances

The Stepwise Regression analysis revealed that, in addition to the influence of sows’ reproductive performances (the litter weight and the number of alive piglets at birth), some specific metabolites can be associated to piglets’ survival and growth parameters (Table 5). In particular, the litter weight at birth and the concentration of acetate significantly entered in the model for LWG (*P* < 0.0001 and *P* = 0.002, respectively); the higher number of alive piglets at birth and the increased concentration of colostrum cis-aconitate were associated with the number of weaned piglets (*P* < 0.0001 and *P* = 0.019, respectively), while dimethylamine (*P* = 0.0002) and taurine (*P* = 0.013) entered as variables in the model for the number of dead piglets per litter at d 3. There was no influence of farrowing season and parity order on LWG, the number of weaned pigs or the number of dead piglets at d 3.

**Table 5:** Results of the Stepwise Regression analysis

Variables	Coefficient	SE coefficient	*T*-value	*P*-value
Model for LWG^a^ [*R*^2^ = 0.4286; C(p) = 0.8735]				
Intercept	0.611	0.540	1.13	0.263
Acetate^b^	0.108	0.033	3.27	0.002
Average piglet’s weight at birth	0.009	0.002	5.39	<.0001
Model for the number of weaned piglets [*R*^2^ = 0.4343; C(p) = 2.0849]				
Intercept	4.801	1.385	12.01	0.001
*cis*- Aconitate^b^	− 0.902	0.372	5.89	0.019
Number of alive piglets at birth	0.584	0.10	35.05	<.0001
Model for the number of piglets dead per litter at d 3 (*R*^2^ = 0.2304; C(p) = 29.1881)				
Intercept	−0.333	0.27	1.44	0.235
Dimethylamine^b^	0.331	0.083	15.82	0.0002
Taurine^b^	− 0.114	0.044	6.64	0.013

^a^LWG stands for the litter body weight gain from birth to d 3

^b^Metabolites concentrations were considered in area arbitrary unit

The outcomes of the Stepwise Regression analysis were then tested with the GLM, and the results reported in Table 6. Both the higher average piglets’ weight at birth (*P* < 0.0001) and the interaction between breed and colostrum acetate concentration (*P* = 0.013) positively affected the LWG (Table 6). The number of alive piglets at birth (*P* = 0.004) and the interaction between cis-aconitate colostrum content and breed (*P* = 0.008) were significantly associated with the number of weaned piglets, while the effect of breed alone presented a trend towards significance (*P* = 0.061). In addition, the number of dead piglets at d 3 was related to breed (*P =* 0.026), to the interaction between the concentration of dimethylamine and breed (*P =* 0.001), to the interaction between taurine concentration and breed (*P =* 0.036) and to the number of alive piglets at birth (*P* = 0.031).

**Table 6:** Results of the GLM analysis

Variables	Coefficient	SE	*P*-value
GLM for LWG^a^			
Intercept	0.892	0.693	0.204
Breed			0.379
LW^c^	0	0	
L^d^	− 1.072	0.934	
D^e^	0.352	0.821	
Acetate^b^ × Breed			0.013
Acetate^b^ × LW^c^	0.094	0.052	
Acetate^b^ × L^d^	0.182	0.062	
Acetate^b^ × D^e^	0.015	0.085	
Average piglet’s weight at birth	0.008	0.002	<.0001
GLM for number of weaned pigs			
Intercept	9.20	1.825	<.0001
Breed			0.061
LW^c^	0	0	
L^d^	− 1.822	1.534	
D^e^	− 6.743	2.85	
*cis*-Aconitate^b^ × Breed			0.008
*cis*-Aconitate^b^ × LW^c^	0.990	1.363	
*cis*-Aconitate^b^ × L^d^	− 0.293	0.596	
*cis*-Aconitate^b^ × D^e^	− 1.616	0.457	
Number of alive piglets at birth	0.341	0.113	0.004
GLM for Number of dead piglets at d 3			
Intercept	−2.226	0.764	0.005
Breed			0.026
LW^c^	0	0	
L^d^	0.190	0.198	
D^e^	−0.271	0.261	
Dimethylamine^b^ × Breed			0.001
Dimethylamine^b^ × LW^c^	−0.271	0.261	
Dimethylamine^b^ × L^d^	0.190	0.198	
Dimethylamine^b^ × D^e^	0.467	0.116	
Taurine^b^ × Breed			0.036
Taurine^b ^× LW^c^	−0.151	0.056	
Taurine^b^ × L^d^	−0.149	0.177	
Taurine^b^ × D^e^	0.174	0.207	
Number of alive piglets at birth	0.127	0.058	0.035

^a^LWG stands for litter body weight gain from birth to d 3 of life

^b^Metabolites concentrations were considered in area arbitrary unit

^c^LW stands for Large White

^d^L stands for Landrace

^e^D stands for Duroc

## Discussion

This is the first study describing in three pig breeds the defatted colostrum metabolome profile < 10 kDa, the factors underlying its composition and the associations between colostrum metabolites and litter’s growth and survival parameters during suckling.

The three breeds showed different reproductive abilities in accordance with the literature [17, 18], with L and LW sows exhibiting a higher average number of piglets alive at birth compared to D sows. These differences between breeds are also visible at the colostrum composition level [19, 20]: considering the whole spectrum, the colostrum composition of L sows showed to be mainly affected by season (explained by PC2), while LW and D breeds displayed clustering tendency for PC1, with the colostrum lactose amount explaining most of the colostrum composition differences between breeds according to [20]. In particular, LW breed samples presented higher values of lactose according to [20]. Lactose concentration in cow milk is commonly associated with the health status of the mammary gland, as higher lactose concentrations are positively correlated to healthier mammary glands and low amounts of this sugar indicate the existence of intramammary infections [21]. Considering the data provided by the present work, it is not possible to support the same association in lactating sows, due to the absence of reference value for sow milk and colostrum.

Furthermore, the obtained colostrum spectra were affected also by the farrowing season: the samples gathered during winter and autumn exhibited differences in colostrum compositions with respect to colostrum secreted during spring and summer. These differences could be ascribed to the environmental conditions affecting sows’ performances. Indeed, even if the sows were reared in temperature-controlled and humidity-controlled environment, heat stress may occur in hotter months and is an important factor that must be taken into account when dealing with results obtained in the Mediterranean areas. Compounds such as acetate, which showed to be more abundant during cold seasons, may reflect the different energy requirements of sows during cold months. Acetate is of particular interest for milk composition as it is a precursor of the fat synthesized in mammary glands [22] and it could be the product of fermentations taking place in sows’ hindgut.

In addition, farrowing season affected also the creatine pathway: in particular, creatine and creatine-phosphate amounts during the period ranging from September to February were significantly lower than in spring and summer; on the contrary, creatinine was higher during the same period. Creatine is an important nutrient for the new-born, as it functions as high-energy phosphate buffer and it is essential in tissues with a high energy demand such as muscle and brain [23]. In mice, it has been shown that milk creatine is extracted from the circulating plasma by the mammary gland, which conversely has little or no capacity to synthesize creatine [24]. No research data are available for sow colostrum, but it can be assumed that also in this case variations in colostrum may reflect variations in blood creatine concentration. Here the variations in the ratio creatine and creatine-phosphate to creatinine may have resulted from a higher degradation of the first two compounds into creatinine during the hotter season. The increasing amount of creatinine level is in general associated with a higher mobilization of stored proteins and indirectly with fat and lean levels in the body mass [25]. A recent study [26] associated an increased amount of blood creatinine on the 1^st^ day of lactation with lower feeding levels in sows during late gestation period. However, we could not control feed intake in the days before farrowing thus we do not have information regarding its variations according to the season. Therefore, further research is necessary to explain variations of creatine and related compounds in colostrum.

Some of the identified compounds were associated with litter weight gain during the first three days of life and to piglets’ survival rates at d 3 and at weaning. In particular, we suppose that the positive effect of acetate on LWG could be linked to the multiple metabolic roles played by this compound, which can be used as energy source by gut mucosa (in particular by colonocytes), as a substrate for the synthesis of cholesterol and long-chain fatty acids [27], and may also stimulate adipogenesis [28]. Additionally, taurine colostrum concentration showed a positive correlation with piglets’ survival rate at three days of life. Taurine was already proven to play a critical role in neonatal development, including the development of the central nervous system and other tissues [29, 30]. Furthermore, taurine represents also an important factor in dietary fat absorption. Indeed, this organic compound is involved in conjugating bile acids, which are extremely important for the absorption of fat in infants [31]. Thus, taurine content in sows’ colostrum may play an essential role for fat digestion and absorption in piglets, similarly to what was observed in humans [32], showing beneficial effects on piglets’ development and energy supply and decreasing the number of dead. As regards the number of dead piglets at three days of life, this performance was positively associated with higher concentration of dimethylamine secreted in colostrum. Dimethylamine is a nitrogenous product, synthesised by bacterial action by the catabolism of trimethylamine or by the metabolism of choline and choline-containing phosphatides. In the literature, dimethylamine was found in sows’ serum [33], and in human milk [34], suggesting that dimethylamine can pass from mother’s serum to milk (and colostrum). Literature is lacking of studies on the effects of dimethylamine in newborn piglets; anyway it is generally accepted that this compound may have genotoxic [35] and irritant effects on mucosae [36], together with lethargy and coordination disorders in animals [37]. Certainly these results refer to prolonged periods of dimethylamine exposure, but it is also reasonable that sows secreting higher contents of dimethylamine in colostrum coupled with the weakness status of piglets at birth could have led to higher numbers of lethargic piglets, resulting in increased losses during the first three days of life.

Similarly to dimethylamine, also cis-aconitate was negatively associated with piglets’ survival capacity from birth to weaning. *cis*-Aconitate is an intermediate of the tricarboxylic acid (TCA) cycle that regulates the energy metabolism and is the result of the reversible isomerization of citrate to isocitrate via M-aconitase enzyme activity [38]. Literature is largely lacking studies about *cis*-aconitate effects in piglets, but in humans found that increased number of fetal malformation cases was associated with higher levels of cis-aconitate in mothers’ serum [39]. Nevertheless, we have not observed foetal malformations in the considered litters. Additionally, another hypothesis can be formulated considering that TCA cycle intermediate metabolites function as metabolic checkpoints for the activation of lipopolysaccharide response genes, such as hypoxia-inducible factor 1-alpha (*HIF1A*), interleukin 1 beta (*IL1B*) and immune responsive gene 1 (*IRG1*) [40]. In particular, in M1 macrophages (that are stimulated for a rapid response against inflammation and pathogens) some breaks in Krebs cycle occur: one of them consists in a redirection of citrate towards the production of itaconic acid (whose intermediate is *cis*-aconitate) [41]. Thus, higher contents of *cis*-aconitate in colostrum may be associated to the existence of immune response in sows, suggesting that also their litters may have been exposed to the same pathogens, causing more deaths in the first three days of life.

## Conclusions

In conclusion, this study demonstrates that colostrum metabolome is greatly affected by breed and, in particular, Duroc sows showed colostrum compositions unlike any other. This result agrees with the generally accepted view that the differences among Duroc and white coated pig breeds may originate from distinct genetic origins, and consequently, suggests that further genetic studies may help to explain the variations found among breeds in colostrum compositions. From the observation of the results obtained it can be suggested that the different temperatures occurring during the year affect sows’ metabolism and, in turn, can also affect colostrum composition. Among the identified metabolites, acetate and taurine showed their positive effects on piglets’ performances from birth to day three of age and on piglets’ survival rate, while dimethylamine and *cis*-aconitate exerted a negative influence on the new-borns’ capacity to survive. This research represents a preliminary step towards the knowledge of pig colostrum composition and it is one of the first studies focusing on the associations between different swine colostrum compositions and litter performances. Further investigations are needed to extend the identification of the different compounds in swine colostrum and to elucidate their effects on new-borns and on piglets during the post-weaning period. Furthermore, the possible interaction between sows’ feeding and microbiota in the modulation of colostrum metabolome deserves further investigations.

## Additional file

Additional file 1: Table S1. The main bins accounting for the spectral differentiation and their relative chemical shift. (DOCX 21 kb)

## Abbreviations

^1^H–NMR: Nuclear magnetic resonance with respect to hydrogen-1 nuclei
ANOVA: Analysis of Variance
ATP: Adenosine Triphosphate
D: Duroc
D_2_O: Deuterated Water
EU: European Union
HOD: form of water in a deuterated solvent environment observable among the ^1^H–NMR spectroscopy peaks
L: Landrace
LBW: Litter Body Weight
LW: Large White
LWG: Litter Weight Gain
PC: Principal Component
PCA: Principal Components Analysis
PQN: Probabilistic Quotient Normalization
SD: Standard Deviation
SE: Standard Error
SEM: Standard Error of the Mean
TMSP: 3-(trimethylsilyl)-propioniate-2,2,3,3-d_4_
UDP: Uridine diphosphate
UMP: Uridine monophosphate

## References

[1] Vallet JL, Calderon-Diaz JA, Stalder KJ, Phillips C, Cushman RA, Miles JR (2016). Litter of origin trait effects on gilt development. J Anim Sci.

[2] Devillers N, Le Dividich J, Prunier A (2011). Influence of colostrum intake on piglet survival and immunity. Animal.

[3] Ferrari CV, Sbardella PE, Bernardi ML, Coutinho ML, Vaz IS, Wentz I (2014). Effect of birth weight and colostrum intake on mortality and performance of piglets after cross-fostering in sows of different parities. Prev Vet Med.

[4] Decaluwé R, Maes D, Wuyts B, Cools A, Piepers S, Janssens GPJ (2014). Piglets’ colostrum intake associates with daily weight gain and survival until weaning. Livest Sci.

[5] Noblet J, Dourmad JY, Etienne M, Le Dividich J (1997). Energy metabolism in pregnant sows and newborn pigs. J Anim Sci.

[6] Rooke JA, Bland IM (2002). The acquisition of passive immunity in the new-born piglet. Livest Prod Sci.

[7] Vallet JL, Miles JR, Rempel LA (2013). A simple novel measure of passive transfer of maternal immunoglobulin is predictive of preweaning mortality in piglets. Vet J.

[8] Vallet JL, Miles JR, Rempel LA, Nonneman DJ, Lents CA (2015). Relationships between day one piglet serum immunoglobulin immunocrit and subsequent growth, puberty attainment, litter size, and lactation performance. J Anim Sci.

[9] Ogawa S, Tsukahara T, Imaoka T, Nakanishi N, Ushida K, Inoue R (2016). The effect of colostrum ingestion during the first 24 hours of life on early postnatal development of piglet immune systems. Anim Sci J.

[10] Schlimme E, Martin D, Meisel H (2000). Nucleosides and Nucleotides: natural bioactive substances in milk and colostrum. Br J Nutr.

[11] Korhonen HJ (2013). Production and properties of health-promoting proteins and peptides from bovine colostrum and milk. Cell Mol Biol.

[12] He Y, Liu S, Leone S, Newburg DS (2014). Human colostrum oligosaccharides modulate major immunologic pathways of immature human intestine. Mucosal Immunol.

[13] Foisnet A, Farmer C, David C, Quesnel H (2011). Farrowing induction induces transient alterations in prolactin concentrations and colostrum composition in primiparous sows. J Anim Sci.

[14] R Core Team. R: a language and environment for statistical Computing 2015. http://www.R-project.org/ . Accessed 11 Jan 2016.

[15] Dieterle F, Ross A, Schlotterbeck G, Senn H (2006). Probabilistic quotient normalization as robust method to account for dilution of complex biological mixtures. Application in ^1^H NMR metabonomics. Anal Chem.

[16] Wu J, Domellöf M, Zivkovic AM, Larsson G, Öhman A, Nording ML (2016). NMR-based metabolite profiling of human milk: a pilot study of methods for investigating compositional changes during lactation. Biochem Biophys Res Commun.

[17] Blasco A, Bindanel JP, Haley CS, Varley MA (1995). Genetic and neonaltal survival. The neonatal pig: development and survival.

[18] Sonderman JP, Luebbe JJ (2008). Semen production and fertility issues related to differences in genetic lines of boars. Theriogenology.

[19] Simmen FA, Whang KY, Simmen RCM, Peterson GA, Bishop MD, Irvin KM (1990). Lactational variation and relationship to postnatal growth of insulin-like growth factor-I in mammary secretions from genetically diverse sows. Domest Anim Endocrinol.

[20] Zou S, McLaren DG, Hurley WL (1992). Pig colostrum and milk composition:comparisons between Chinese Meishan and US breeds. Livest Prod Sci.

[21] Park YK, Koo HC, Kim SH, Hwang SY, Jung WK, Kim JM (2007). The analysis of milk components and pathogenic bacteria isolated from bovine raw milk in Korea. J Dairy Sci.

[22] Linzell JL, Mepham TB (1969). Mammary metabolism in lactating sows: arteriovenous differences of milk precursors and the mammary metabolism of [14C]glucose and [l4 C]acetate. Br J Nutr.

[23] Brosnan JT, Brosnan ME (2007). Creatine: endogenous metabolite, dietary, and therapeutic supplement. Annu Rev Nutr.

[24] Lamarre SG, Edison EE, Wijekoon EP, Brosnan ME, Brosnan JT (2010). Suckling rat pups accumulate creatine primarily via de novo synthesis rather than from dam milk. J Nutr.

[25] Van Niekerk BD, Reid JT, Bensadoun A, Paladines OL (1963). Urinary creatine as an index of body composition. J Nutr.

[26] Decaluwé R, Maes D, Cools A, Wuyts B, De Smet S, Marescau B (2014). Effect of peripartal feeding strategy on colostrum yield and composition in sows. J Anim Sci.

[27] Den Besten G, Lange K, Havinga R, Van Dijk TH, Gerding A, Van Eunen K (2013). Gut-derived short-chain fatty acids are vividly assimilated into host carbohydrates and lipids. Am J Physiol Gastrointest Liver Physiol.

[28] Hong YH, Nishimura Y, Hishikawa D, Tsuzuki H, Miyahara H, Gotoh C (2005). Acetate and propionate short chain fatty acids stimulate adipogenesis via GPCR43. Endocrinology.

[29] Bryson JM, Jackson SC, Wang H, Hurley WL (2001). Cellular uptake of taurine by lactating porcine mammary tissue. Comp Biochem Physiol - B Biochem Mol Biol.

[30] Aerts L, Van Assche FA (2002). Taurine and taurine-deficiency in the perinatal period. J Perinat Med.

[31] Gaull GE (1989). Taurine in pediatric nutrition: review and update. Pediatrics.

[32] Guilloteau P, Zabielski R, Hammon HM, Metges CC (2010). Nutritional programming of gastrointestinal tract development. Is the pig a good model for man?. Nutr Res Rev.

[33] He Q, Kong X, Wu G, Ren P, Tang H, Hao F (2009). Metabolomic analysis of the response of growing pigs to dietary L-arginine supplementation. Amino Acids.

[34] Lichtenberger LM, Gardner JW, Barreto JC, Morriss FH (1991). Evidence for a role of volatile amines in the development of neonatal hypergastrinemia. J Pediatr Gastroenterol Nutr.

[35] Galli A, Paolini M, Lattanzi G, Cantelli-Forti G, Bronzetti G (1993). Genotoxic and biochemical effects of dimethylamine. Mutagenesis.

[36] Fluhr JW, Kelterer D, Fuchs S, Kaatz M, Grieshaber R, Kleesz P (2005). Additive impairment of the barrier function and irritation by biogenic amines and sodium lauryl sulphate: a controlled in vivo tandem irritation study. Skin Pharmacol Physiol.

[37] Dimethylamine. In: The MAK Collection for Occupational Health and Safety. 2012. http://onlinelibrary.wiley.com/doi/10.1002/3527600418.mb12440e0007/full. Accessed 22 Jan 2018.

[38] Cantu D, Schaack J, Patel M (2009). Oxidative inactivation of mitochondrial aconitase results in iron and H2O2-mediated neurotoxicity in rat primary mesencephalic cultures. PLoS One.

[39] Diaz SO, Pinto J, Graça G, Duarte IF, Barros AS, Galhano E (2011). Metabolic biomarkers of prenatal disorders: an exploratory NMR metabonomics study of second trimester maternal urine and blood plasma. J Proteome Res.

[40] Papathanassiu AE, Ko JH, Imprialou M, Bagnati M, Srivastava PK, Vu HA (2017). BCAT1 controls metabolic reprogramming in activated human macrophages and is associated with inflammatory diseases. Nat Commun.

[41] O’Neill LA (2015). A broken krebs cycle in macrophages. Immunity.

