# Supplementary material for: Metabolomics characterization of colostrum in three sow breeds and its influences on piglets’ survival and litter growth rates
Source: J Anim Sci Biotechnol. 2018 Mar 7;9:23. doi: 10.1186/s40104-018-0237-1 (PMC5840723; doi:10.1186/s40104-018-0237-1)
Supplement: Supplementary file 1 — The main bins accounting for the spectral differentiation and their relative chemical shift. (DOCX 21 kb) [file 40104_2018_237_MOESM1_ESM.docx]

Additional file 1: Table S1. The main bins accounting for the spectral differentiation and their relative chemical shift.

|  | Bin  Number | ppm  Interval | Metabolite^I^ | Matabolite Name |
| --- | --- | --- | --- | --- |
| PC1 | 10-11 | 8.134-8.071 | 25 | UMP |
|  | 15 | 7.980-7.936 | 20 and 22 | UDP- glucose, UDP- galactose. |
|  | 27-29 | 6.026-5.939 | 6, 22 and 23 | UDP-N-Acetylglucosamine, UDP- galactose and Uridine |
|  | 39-41 | 5.675-5.588 | 22 | UDP- galactose |
|  | 43-45 | 5.587-5.500 | 6 | UDP-N-Acetylglucosamine |
|  | 49 | 5.412-5.368 | NA |  |
|  | 54-56 | 5.280-5.192 | 19 | Lactose |
|  | 58-60 | 5.192-5.083 | NA |  |
|  | 67-70 | 4.507-4.397 | 19,20 and 22 | Lactose, UDP- glucose and UDP- galactose |
|  | 72-75 | 4.396-4.288 | 22 | UDP- galactose |
|  | 77-84 | 4.287-4.090 | 18 and 22 | Myo-Inositol and UDP- galactose |
|  | 86-108 | 4.090-3.475 | 17,18,19 and 20 | Taurine, Myo-Inositol, Lactose and UDP- glucose |
|  | 110-112 | 3.475-3.388 | NA |  |
|  | 114-123 | 3.387-3.146 | 7,16,17,18 and 19 | O-Acetylcholine, sn-Glycerophosphocholine, Taurine, Myo-Inositol and Lactose |
|  | 127-128 | 3.080-3.0149 | 11,12 and 13 | Creatine, Creatinine phosphate and Creatinine |
|  | 135 | 2.839-2.795 | NA |  |
|  | 137-139 | 2.795-2.770 | 10 | Dimethylamine |
|  | 141-143 | 2.707-2.619 | 9 | Citrate |
|  | 146-147 | 2.578-2.510 | 9 | Citrate |
|  | 165 | 2.114-2.071 | NA |  |
|  | 167-168 | 2.070-2.005 | NA |  |
|  | 174-176 | 1.851-1.763 | NA |  |
|  | 184-185 | 1.367-1.3021 | 1 | Lactate |
|  | 198 | 0.973-0.929 | NA |  |
| PC2 | 2 | 8.485-8.441 | 24 | Formate |
|  | 7 | 8.221-8.178 | NA |  |
|  | 9-11 | 8.156-8.068 | 25 | UMP |
|  | 13-16 | 8.024-7.914 | 6,20 and 22 | UDP-N-Acetylglucosamine, UDP- glucose and UDP- galactose |
|  | 27-29 | 6.026-5.939 | 6,22 and 23 | UDP-N-Acetylglucosamine, UDP- galactose and Uridine |
|  | 33-34 | 5.895-5.807 | NA |  |
|  | 39-46 | 5.674-5478 | 6 and 22 | UDP-N-Acetylglucosamine and UDP- galactose |
|  | 53-56 | 5.302-5.192 | 19 | Lactose |
|  | 59 | 5.170-5214 | NA |  |
|  | 64 | 4.973-4.929 | NA |  |
|  | 68-73 | 4.485-4.331 | 22 | UDP- galactose |
|  | 75-122 | 4.331-3.168 | 7,16,17,18,19,20,21 and 22 | O-Acetylcholine, sn-Glycerophosphocholine, Taurine, Myo-Inositol, Lactose, UDP- glucose, Glycolate and UDP- galactose |
|  | 124-125 | 3.168-3.102 | 14 and 15 | cis-Aconitate and Choline |
|  | 127-128 | 3.080-3.014 | 11,12 and 13 | Creatine, Creatinine phosphate and Creatinine |
|  | 130 | 3.014-2.971 | NA |  |
|  | 135 | 2.839-2.795 | NA |  |
|  | 137-139 | 2.795-2.770 | 10 | Dimethylamine |
|  | 141-143 | 2.707-2.619 | 9 | Citrate |
|  | 146-148 | 2.578-2.488 | 9 | Citrate |
|  | 163 | 2.1587-2.114 | NA |  |
|  | 165 | 2.114-2.071 | NA |  |
|  | 167-168 | 2.070-2.005 | NA |  |
|  | 171-172 | 1.9612-1.895 | 3 | Acetate |
|  | 174-176 | 1.851-1.763 | NA |  |
|  | 184-189 | 1.367-1.193 | 1 | Lactate |
|  | 194 | 1.127-1.083 | NA |  |
|  | 198-199 | 0.973-0.907 | NA |  |

^I^ Metabolite assigned number referred to Table 2.
